# Patients’ Expectations and Perspectives on Follow-up Care after Bariatric Surgery in Germany

**DOI:** 10.1007/s11695-025-07890-w

**Published:** 2025-04-30

**Authors:** Jonas Wagner, Madita Roll, Anne Lautenbach, Sara Notz, Gabriel Plitzko, Jakob Izbicki, Oliver Mann, Thilo Hackert, Anna Duprée, Freya Brodersen, Angelika Weigel

**Affiliations:** University Medical Center Hamburg-Eppendorf, Hamburg, Germany

**Keywords:** Bariatric surgery, Obesity, Follow Up care, Expectations, Outcomes

## Abstract

**Background:**

Bariatric surgery is the most effective treatment for patients with obesity. After surgery, lifelong follow-up care is recommended to improve weight-loss outcomes. However, follow-up attendance is low, and the reasons have yet to be determined. Therefore, the present study aimed to identify patients’ expectations and perspectives on follow-up care after bariatric surgery to identify current unmet needs and ways to increase follow-up attendance.

**Methods:**

Patients who underwent bariatric surgery at a university medical center and attended at least one follow-up appointment completed an online questionnaire. The questionnaire consisted of open-ended questions regarding follow-up care. Content analysis was applied to qualitatively analyze the results.

**Results:**

In total, 164 patients responded to at least one question (participation rate 50.1%). On average, patients had attended three follow-up appointments at the time of the study. Expectations concerning the content of follow-up care included regular examinations, blood tests, and psychological and nutritional counseling and support. Notably, the follow-up care was most criticized for its lack of regular psychological and nutritional support, with many suggesting that these services be incorporated. Interestingly, follow-up care appointment attendance was rarely connected with expectations of better outcomes. Regular appointments and laboratory results were among the positive aspects of the current follow-up care.

**Conclusions:**

Expectations were mostly related to the content of follow-up care. However, few patients seemed to recognize the importance of follow-up care for improved outcomes. Therefore, educating patients about the critical role of follow-up care might improve attendance and also lead to more successful long-term bariatric surgery outcomes.

**Supplementary Information:**

The online version contains supplementary material available at 10.1007/s11695-025-07890-w.

## Introduction

Obesity is associated with significant morbidity and mortality. The prevalence of obesity has tripled since 1975, making obesity one of the leading challenges for health systems worldwide [1–3]. Bariatric surgery has been established as the most effective treatment for obesity and its comorbidities, for example type 2 diabetes or cardiovascular disease [4–8]. In Germany, patients are eligible for bariatric surgery if their body mass index (BMI) exceeds 40 kg/m^2^ or if it is above 35 kg/m^2^ in the presence of obesity-related comorbidities such as type 2 diabetes, hypertension, cardiovascular disease, or sleep apnea in accordance with the German Guidelines of the Surgical Treatment of Obesity [9]. Patients should be monitored after bariatric surgery to detect possible complications such as nutritional deficiencies and improve outcomes after surgery [10]. Ideally, the loss to follow-up rate should be less than 20% [11]. However, the attendance rate after bariatric surgery decreases continuously, and usually less than 50% of patients still attend follow-up care appointments a few years after surgery [12–18]. Young age, male sex, an avoidant relationship style, and unemployment have been identified as factors associated with lower follow-up care appointment attendance [19–21], whereas results regarding distance to the center are conflicting [22, 23]. Guidelines recommend lifelong follow-up after surgery to ensure optimal outcomes, which include sustained weight loss and the early detection and management of complications such as nutritional deficiencies [9, 24]. Evidence indicates that regular follow-up enhances weight-loss outcomes [25, 26]. However, the specific content of follow-up care that drives these improvements remains unclear. Therefore, identifying starting points to improve attendance rates is crucial for improving bariatric surgery outcomes.

One approach to improve treatment outcomes is by managing patient expectations. Expectations can be defined as specific beliefs regarding the probability of subsequent events [27, 28], such as the course of the disease, treatment outcomes, the probability of complications or side effects and the capability to influence the occurrence of complications or side effects. In a randomized controlled trial, a brief psychological intervention to manage patient expectations before coronary artery bypass graft surgery improved outcomes [29, 30]. Furthermore, positive expectations were associated with better outcomes in patients undergoing surgery, those experiencing pain, fatigue, and muscular disorders, and those receiving psychological treatments [31–37].

To our knowledge, no previous studies have examined the relationship between patient expectations regarding follow-up care and subsequent attendance or outcomes in the context of bariatric surgery. Recent studies reported that more research is needed for the development of interventions aimed at enhancing adherence and improving patient outcomes [38–40]. Our study aims to fill this gap by delineating the expectations of patients in Germany, thereby providing novel insights that may inform future strategies to improve follow-up adherence and overall outcomes. These findings are crucial for designing patient-centred healthcare services, by identifying patients’ expectations of post-bariatric surgery follow-up care, assessing their views on the current program’s strengths, and gathering their recommendations for improvements and solutions to existing challenges.

## Materials and Methods

### Sampling Method and Procedure

We recruited patients by phone who had undergone bariatric surgery at our Center of Excellence for Bariatric Surgery and met the following inclusion criteria: adults (18 years or older), surgery performed between April 2016 and December 2022, attendance of at least one follow-up appointment, fluency in German, access to the internet, and willingness to participate in our biobank (PV4889) and this study. These calls were conducted by three of the authors (J. W., M. R., and F. B.), and the patients received a brief explanation of the study’s purpose, procedures, and the importance of their input. This was a convenience sample, as participants were selected based on their availability and willingness to participate. Patients willing to participate received a link to the online questionnaire (LimeSurvey; Version 5) (Table 1). The institutional review board approved the biobank and study, and all patients provided informed consent.

**Table 1:** Online questionnaire translated from German

Online questionnaire	
Question 1	What are your expectations regarding follow-up care after bariatric surgery?
Question 2	What are the positive aspects of the follow-up care program?
Question 3	What can be improved in the current follow-up care program?
Question 4	What is currently missing in the program?

### Follow-up Program

The follow-up program at our Center of Excellence for bariatric surgery was carried out in accordance with German guidelines and fully covered by statutory health insurance [9]. In brief, follow-up examinations and laboratory checks were scheduled for 3, 6, 12, 18, and 24 months post-surgery, and annually thereafter. Appointments for the first 2 years post-surgery were scheduled for patients at hospital discharge, and the appointments afterward were scheduled by each patient individually. Examinations were performed by either a bariatric surgeon or an endocrinologist experienced in bariatric surgery and included a medical history assessment, physical examination, weight and waist circumference measurements, and laboratory checks. Depending on the patient’s expressed needs, professionals in other specialties, such as nutrition counselors or psychosomatic care providers, were involved.

### Data Collection and Analysis

Expectations of health care are diverse [41]; therefore, a qualitative research approach was chosen to capture a broad range of patients’ expectations and perspectives on their current follow-up care. We designed this study around four open-ended questions using an online questionnaire (Table 1). Through the use of the questionnaire, we aimed to increase the number of participants, as larger sample sizes in quantitative research typically offer advantages such as greater generalizability, enhanced reliability and stability, and improved external validity [42–44]. Additionally, the questionnaire is standardized, making biases introduced by interviewers or other participants irrelevant [45]. Also, studies investigating the patients’ perspectives using interviews, semi-structured interviews or focus groups have already been performed [46–50].

Our main focus in this study was to investigate patient expectations for the follow-up care to understand whether expectation management could be used to improve follow-up care adherence. Additionally, we sought to understand which parts of the current follow-up care were well received by patients to identify important parts that should continue to be provided. Patients were also asked to make suggestions about potential improvements as well as to point out shortcomings in their follow-up care. The responses to the four open-ended questions were qualitatively analyzed using content analysis based on Mayring’s approach, as this method allows for a systematic assessment of qualitative data and is applicable to various types of content [51]. Content analysis was performed independently by two authors (J. W. and M. R.). In line with recommendations for content analysis, we conducted an iterative process of reading and coding the qualitative data, during which text segments were initially assigned to preliminary categories. The categories were then defined in a codebook to categorize the responses (Supplementary file 1). Regular discussions among the research team ensured that the categories were refined and that consensus was reached on their definitions and boundaries. Throughout this process, we maintained reflexivity by continuously examining our own assumptions and biases, further enhancing the credibility and depth of our analysis. Categories were split into main and secondary categories. The dataset was screened again, and the responses were subsequently assigned to the defined main and secondary categories. In order to capture both in-depth insights and quantitative trends, we deliberately applied a mixed methods approach. Following the qualitative analysis, the main and secondary categories were then counted to detect the frequency of each, thereby integrating rich, context-driven interpretations with numerical data to enhance the overall rigor of the study. Examples of the analysis process are provided in Supplementary file 2.

Data on anthropometric characteristics (height, weight, body mass index (BMI)), comorbidities (type 2 diabetes (T2D), hypertension, obstructive sleep apnea (OSA), dyslipidemia, depression, anxiety disorder), and type of surgery were collected as part of our biobank. We performed statistical analysis with the Statistical Package for Social Sciences software (SPSS; IBM, Version 24). Patient characteristics are presented as the *mean* ± standard deviation (*SD*) for continuous variables. For comparisons between continuous variable groups, independent Student’s *t*-test was performed. We used the *χ*^2^ test to analyze differences between nominal data.

## Results

### Patient Characteristics

Overall, 327 patients were contacted, 164 patients of whom responded to at least one question; these patients were included in the qualitative analysis (participation rate 50.1%). The patients had a mean age of 44 ± 11.1 years, and most were female (221/68%). The average preoperative weight was 151 kg ± 27.1 kg, and the mean BMI was 50.5 ± 7.9 kg/m^2^. The procedures included laparoscopic sleeve gastrectomies, Roux-en-Y gastric bypass (RYGB), and others. At the time of the study, patients had attended an average of 3.3 follow-up care appointments (Table 2), resulting in a follow-up rate of 72.5%. The longest available follow-up was 6 years, and the shortest was 3 months. The majority of patients were in the first (25.7%), second (32.7%), or third year (32.4%) following their surgery. The characteristics of patients who responded were not significantly different from those who did not respond (Table 3).

**Table 2:** Preoperative patient characteristics and average follow-up appointments

Characteristic	
Contacted patients [*n*]	327
Age [years ± SD]	44 ± 11.1
Weight [kg﻿ ± SD]	151 ± 27.1
Height [cm﻿ ± SD]	173 ± 9.5
BMI [kg/m^2﻿^﻿ ± SD]	50.5 ± 7.9
Sex [*n* female/%]	221/67.6
Average attended follow-up appointments [*n﻿ ± SD*]	3.3 ± 1.3
Bariatric surgery procedure [*n*/%]	
• Sleeve gastrectomy	256/78.3
• RYGB	65/19.9
• Others	6/1.8
Type 2 diabetes [*n*/%]	100/30.6
Hypertension [*n*/%]	177/54.1
OSA [*n*/%]	71/21.7
Dyslipidemia [*n*/%]	166/50.8
Depression [*n*/%]	52/15.9
Anxiety disorder [*n*/%]	11/3.4

**Table 3:** Preoperative characteristics of responders and non-responders and average follow-up appointments

Characteristic	Responders	Non-responders	*p* value
Patients [*n*]	164	163	
Age [years ﻿± SD]	45 ± 11.3	43 ± 10.8	0.05
Weight [kg﻿ ± SD]	149 ± 26.4	153 ± 27.6	0.15
Height [cm﻿ ± SD]	173 ± 8.8	174 ± 10.1	0.51
BMI [kg/m^2^ ± SD]	50.1 ± 8.2	51 ± 7.6	0.3
Sex [*n* female/%]	117/71.3	104/63.8	0.15
Average attended follow-up appointments [*n﻿ ± SD*]	3.3 ± 1.4	3.2 ± 1.3	0.2
Bariatric surgery procedure [*n*/%]			
• Sleeve gastrectomy	124/75.6	256/81	0.28
• RYGB	38/23.2	27/16.6	0.28
• Others	2/1.2	4/2.5	0.28
Type 2 diabetes [*n*/%]	50/30.5	50/30.7	0.61
Hypertension [*n*/%]	94/57.3	83/50.9	0.25
OSA [*n*/%]	38/23.2	33/20.2	0.52
Dyslipidemia [*n*/%]	79/48.2	87/53.4	0.35
Depression [*n*/%]	21/12.8	31/19	0.12
Anxiety disorder [*n*/%]	4/2.4	7/4.3	0.35

### Patients’ Expectations for Follow-up Care After Bariatric Surgery

We received responses from 141 patients regarding their expectations, resulting in a response rate of 43%. We identified four categories, namely structural aspects, advice, support and outcome.

#### Expectations Towards Structural Aspects of the Follow-up Program

**Fig. 1:**
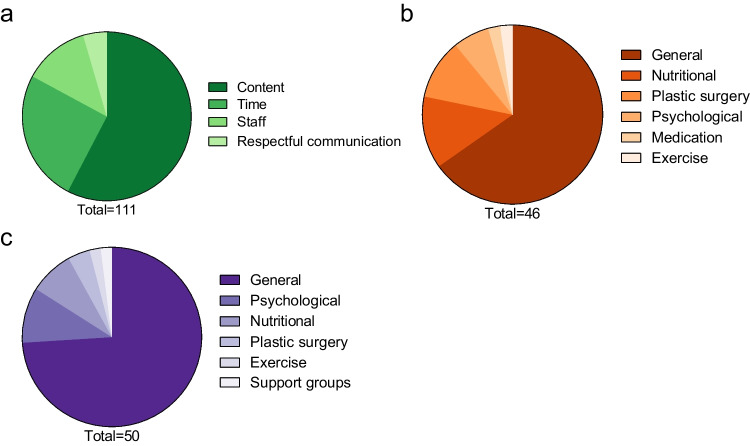
Proportions of subcategories for structural components (**a**), advice (**b**), and support (**c**) for question one. Figures below the pie chart indicate the number of responses for each category

Patients’ expectations regarding structural aspects of the program concerned the content, time, staff, and respectful communication. The most frequent responses were related to the content of the follow-up program (Fig. 1A), with a particular emphasis on the importance of regular blood tests. Many participants highlighted the need for ongoing monitoring to ensure their health and detect potential deficiencies early. For example, one participant stated: “It is important for me to check my blood values to make sure that there are no deficiency symptoms” (patient 46, female). Another patient also emphasized the importance of regular blood test for her: “Regular blood tests are very important to me because general practitioners often refuse to conduct such extensive evaluations, as they are not sufficiently familiar with the topic of bariatric surgery.” (patient 146, female). This reflects a strong concern among patients about the long-term effects of bariatric surgery on nutritional health.

Other expectations concerned the timing of follow-up care and the desire for an individualized approach: “Competent medical check-ups at regular intervals and be prepared to answer specific individual questions” (patient 31, female). Additionally, patients expressed a desire for tailored follow-up care that considers their unique health profiles and post-surgical experiences. One participant remarked: “Regular check-ups and consultations. Feeling that you’re not just a number being ticked off a list.” (patient 51, female). These responses highlight the importance of designing follow-up care that respects patients’ individual circumstances while also providing timely and accessible support.

Additionally, patients also expressed expectations regarding the staff: “Flexible, reliable and prompt contact partners” (patient 40, female). Patients also underscored the value of a specific contact person throughout their follow-up care: *“*That a contact person is still available even after a long time and that help is provided at any time” (patient 142, female). This highlights the importance of the staff during the patients’ post-surgical journey.

Last, patients expected respectful and empathic communication: “I find it important that my worries and problems are taken seriously and that I get help when I need it” (patient 38, female). According to patients, respectful and empathic communication is the basis for building a positive relationship and maintaining adherence to the program.

#### Expectations Towards Advice

The next category identified was advice. We defined advice as information, guidance, or explanations. Responses regarding advice could be divided into six subcategories: general, nutritional, plastic surgery, psychological, medication, and exercise advice (Fig. 1B). Most patients expressed general advice expectations, for example, “Good advice, suggestions, point out ideas” (patient 117, male). Although most patients mentioned general advice, it is challenging to draw definitive conclusions from these responses. Specific categories, such as nutritional counseling, were more insightful, which provided greater depth and clarity. One patient stated: “Tips on nutritional issues (quantities, tolerances, intolerances). Which dietary supplements are necessary*?*” (patient 113, female). Another patient responded: “Additional nutritional counseling or tailored training plans for everyday life, especially for a stressful work routine. This specific aspect needs to be addressed more intentionally” (patient 222, male). These responses highlight the importance of nutritional counseling after surgery. Advice regarding plastic surgery was also mentioned by some patients: “Advice on excess skin after weight loss” (patient 164, female). Another patient remarked: “Information about further steps in case of successful weight loss, e.g., body contouring procedures” (patient 222, male). Additionally, patients expected psychological advice to be provided: “A little more psychological advice” (patient 22, female).

#### Expectations Towards Support

The next category that was identified was support. We defined support as assistance in achieving or acting on goals that included general, psychological, nutritional, or plastic surgery support; support groups; and exercise (Fig. 1C). Like with advice, most patients had general support expectations. For instance, one patient said, “I expect assistance to continue successful weight loss” (patient 129, female). The more specific subcategories offered deeper and more meaningful insights. Patients expected psychological support: “My expectation would be that my psyche would be straightened out, my depression treated and my significant eating disorders eliminated” (patient 23, male). Eating disorders were also mentioned by another patient: “Support for eating disorders” (patient 137, male). Like in the advice category, patients also expected nutritional support: “Additional support with nutrition plans and uncovering your own mistakes that you are unaware of” (patient 77, male). Some patients shared expectations, which we categorized as individual cases, for example, “I did not have expectations” (patient 110, female).

#### Expectations Towards Outcome

Finally, only two patients expected that attending follow-up care is associated with a better outcome: “That regular control leads to a better result” (patient 28, male) and “That it leaves me healthy and balanced*”* (patient 260, male).

### Patients’ Perspective on Follow-up Care After Bariatric Surgery

**Fig. 2:**
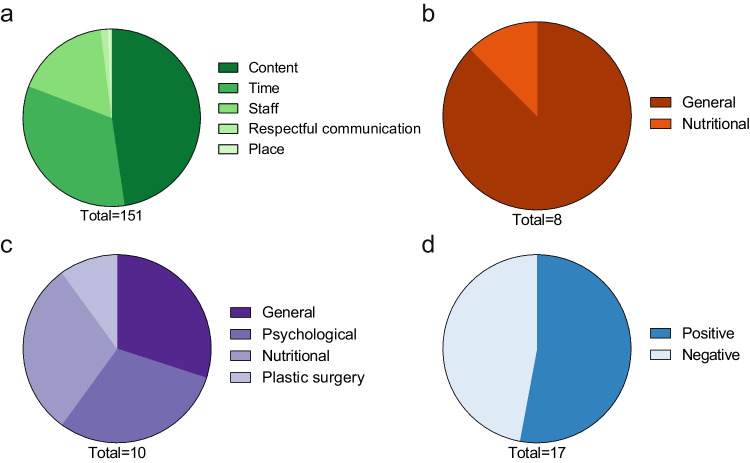
Proportions of subcategories for structural components (**a**), advice (**b**), support (**c**), and personal experiences (**d**) for question two. Figures below the pie chart indicate the number of responses for each category

**Fig. 3:**
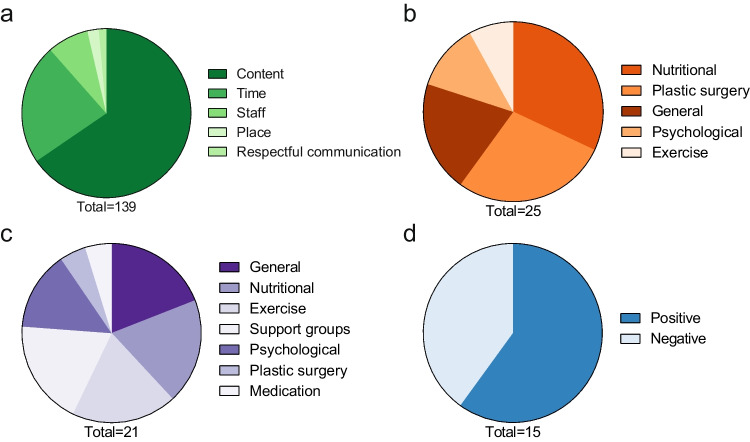
Proportions of subcategories for structural components (**a**), advice (**b**), support (**c**), and personal experiences (**d**) for question three. Figures below the pie chart indicate the number of responses for each category

**Fig. 4:**
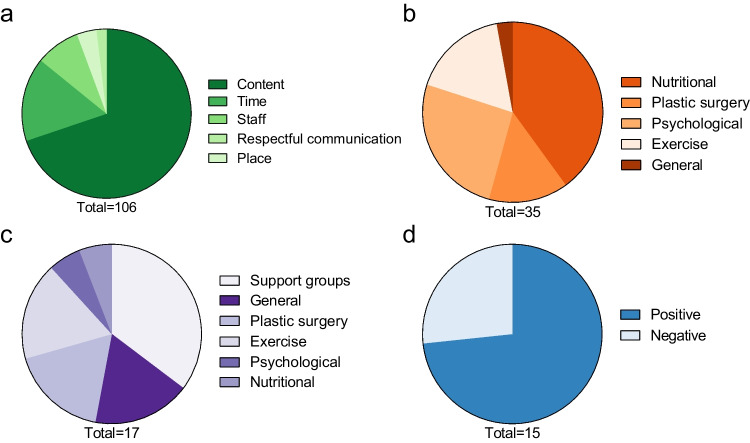
Proportions of subcategories for structural components (**a**), advice (**b**), support (**c**), and personal experiences (**d**) for question four. Figures below the pie chart indicate the number of responses for each category

We received responses from 140 patients regarding positive aspects, 136 patients regarding improvements, and 129 patients regarding shortcomings of their current program, yielding response rates of 43%, 41%, and 39%, respectively. We identified four main categories related to the patients’ perspectives on follow-up care: structural aspects, advice, support, and personal positive and negative experiences. In addition, secondary categories were identified, and their proportions were calculated for positive aspects (Fig. 2A–D), improvements (Fig. 3A–D), and shortcomings (Fig. 4A–D).

#### Patients’ Perspective on Structural Aspects of the Follow-up Program

The patients’ perspective on structural aspects of the follow-up program encompassed factors such as content, time, staff, respectful communication, and place. The most frequent comments were related to the content of the follow-up program. For example, one patient commented, “Checking the current (lab) values. Talk with a doctor” (patient 128, male). Additionally, patients praised the regularity of the appointments: “Fixed dates for the next few years, easy to plan in advance” (patient 4, female). Although patients were satisfied with the regularity of the appointments, long waiting times were mentioned as a negative aspect of the program: “The waiting times were long, despite having an appointment” (patient 9, female). For improvements, one patient suggested that the program should “offer building blocks. Every individual is different. One needs a lot of help with nutrition. The next one needs help with sport, etc. *…*” (patient 22, female). Additionally, one patient suggested, “The psychological component should be part of the follow-up care. Perhaps simply as a standardized component, at least in the first year” (patient 108, female). Moreover, some patients requested the inclusion of an online application in their follow-up care. Other patients were not satisfied with the current follow-up program, for example: “I had to organize everything else myself. Not good.” (patient 191, female).

#### Patients’ Perspective on Advice

The patients’ perspective on advice in the follow-up program included aspects such as nutritional, plastic surgery, general, and psychological and exercise advice. On the positive side, one patient commented, “The medical consultation in which one is further enlightened and receives tips” (patient 145, male). However, some patients missed or wished for more additional advice. For instance, one patient commented, “I would also like to have received regular nutritional advice after the operation, which could be supplemented by cooking courses” (patient 295, female). Another patient commented on what was missing in the current program, “Psychological advice and personal discussion of the results as well as nutritional advice” (patient 183, female). Patients also shared positive experiences regarding their journey after bariatric surgery. For example, one patient expressed satisfaction with the current follow-up program “I can hardly answer this question as my weight reduction is going well. I feel that I am in good hands and all my questions are answered” (patient 222, male).

#### Patients’ Perspective on Support

The patients’ perspective on support in the follow-up program included topics such as general support, psychological support, nutritional support, exercise support, plastic surgery support, support groups, and medication support. Seven patients were satisfied with the support they received during follow-up. For instance, one patient commented, “Opportunity to talk to the nutritionist. Possibility to get referrals for plastic surgery or psychosomatic.” (patient 161, female). Dissatisfaction with the support received during follow-up care was also expressed. For example, one patient complained, “I didn't have any psychological support after the operation, and the nutritional advice was also rather problematic in my case. I don't need someone to explain the food pyramid to me. I need someone to work with me on my behavior” (patient 159, male). Some patients suggested that support be provided through self-support groups. For example, one patient proposed “Regular exchanges with other patients” (patient 196, female).

## Discussion

The present study aimed to identify expectations regarding follow-up care in patients who underwent bariatric surgery as well as their views on their current follow-up care program. Patients predominantly expressed general expectations related to the content of the follow-up program, such as clinical and blood examinations. Furthermore, patients stressed their expectations regarding the time aspects of follow-up care, such as regular appointments and waiting times. The German guidelines recommend lifelong follow-up after bariatric surgery, which should include examinations and laboratory checks. These expectations, which were mentioned by our patients, are in line with the current follow-up and German guidelines [9]. The expressed expectations might be interpreted as patients believing that follow-up care is predominantly necessary for detecting complications and nutritional deficiencies. However, further research is necessary to confirm this phenomenon.

According to our patients, examinations, laboratory checks and regular appointments were mentioned as positive aspects of the current program. In another study, patients noted that regular monitoring of important parameters, such as weight and blood test, was a positive aspect of the program [50]. In particular, the regularity of the appointments was mentioned positively. On the other hand, long waiting times were considered negative structural aspects of the current program. This is often the result of a high load of patients attending follow-up appointments. The number of bariatric procedures is increasing and with a recommended lifelong follow-up, there is an ever-increasing number of patients needing follow-up care [52, 53]. Therefore, new modern methods of follow-up care need to be developed to address the growing number of patients. According to our data, some patients suggested the use of an online application in follow-up programs. Such a tool could help address the growing patient population by enhancing accessibility to follow-up care, facilitating timely monitoring, boosting patient engagement, reducing costs, and promoting patient-centered care. The application should replicate the full spectrum of in-person follow-up care to ensure that patients receive the same support remotely. For instance, in the BELLA Pilot Trial, standardized questionnaires were automatically sent to participants’ smartphones every 6 weeks. In cases where responses were delayed or raised concerns, automated warning messages were delivered to healthcare professionals, and laboratory results could be transmitted directly to the bariatric center for timely review and intervention. [54, 55]. Additionally, a recent meta-analysis reported that digital health applications seem feasible and safe with potential benefits for the digitalization of the postoperative care; however, additional research is needed in this regard [56].

Interestingly, only two patients expected that attending follow-up care appointments would lead to a better result after surgery. Attending follow-up care appointments after bariatric surgery is associated with a better outcome [10, 26]. According to our data, most patients seem to be unaware of this fact. Similar studies did not report that patients recognized that attending follow-up care leads to better weight-loss outcomes [46–48, 50, 57–59]. Therefore, patients should be educated about the importance of follow-up care. Interestingly, patients tend to overestimate weight loss after surgery, which can lead to disappointment [60]. This also implies that there is a need for patient education in the field of bariatric surgery. Recent literature shows that managing expectations prior to an intervention can impact treatment outcomes. The randomized controlled PSY-HEART trial showed that in the field of surgery, expectation management improved the length of stay and long-term outcomes such as disability 6 months after surgery [29, 30]. Therefore, patients would benefit from a repeated delivery of psychoeducation on the importance of follow-up care before and after surgery. However, long-term data regarding expectation management and outcomes are lacking in the field of bariatric surgery. Ultimately, additional research could investigate whether managing expectations leads to improved follow-up adherence after bariatric surgery and, consequently, to improved patient outcomes.

The majority of patients’ expectations and perceptions centered around the structure of the follow-up care program, while a smaller proportion expected to receive specific advice. Patients expected advice regarding nutrition, plastic surgery, psychology, medication, and exercise. Only a few patients mentioned advice as a positive aspect of the current program. Therefore, as patients expected to receive advice, patient expectations are unlikely to be met in this regard. As outlined above, unmet expectations can lead to disappointment [60]. Additionally, if patients do not expect their needs to be addressed, they will not attend follow-up appointments [61]. One solution to improve follow-up appointment attendance might be found in personalized treatment approaches where patients’ needs are assessed, and follow-up care is structured around those individual needs. In their suggestions for improvements, patients were specific: one patient suggested offering a modular follow-up care so that patients could choose which elements to include in their follow-up. Psychological and nutritional advice and advice regarding plastic surgery were also mentioned by patients. Psychological needs have already been reported in the literature [46–48, 57–59]. For example, in this and other studies, bariatric surgery patients reported that psychological support is needed for poor body image, eating disorders or depression [57]. Preoperative depression does not affect the outcome of bariatric surgery, but postoperative depression is associated with poorer outcomes [62–64]. Additionally, some studies have reported that there is an increased suicide rate in patients who undergo bariatric surgery [65–69]. Therefore, evaluating the psychological needs of patients should be performed regularly during follow-up care, and patients should be referred for psychotherapeutic care if needed. However, a quantitative assessment of psychological needs, for example with an adjusted supportive care needs survey, during follow-up care after bariatric surgery has yet to be reported, and this information could help to identify the extent of psychological support needed. Overall, additional research is needed to quantify patient needs during follow-up and to specifically address these needs. Similar to their expectations for advice, patients expected psychological, nutritional, plastic surgery, and exercise support as well as support groups. Only a few patients mentioned support as a positive aspect of their follow-up care. In their suggestions, patients again highlighted the need for psychological, nutritional, and exercise support as well as support groups. A systematic review reported that social support was associated with greater weight loss after surgery [70]. However, the involvement of support groups in follow-up care was mentioned by only six patients, potentially indicating that the benefits of such groups may not be widely recognized among patients. Therefore, steps should be taken to increase patient awareness and actively integrate support groups into follow-up appointments, which could help patients achieve improved weight-loss outcomes.

The strengths of this study include the large number of patients and the inclusion of patients at different time points after surgery. Limitations include the single-center design, rare expectations that could have been missed, and the relatively short responses due to the use of an online questionnaire. Another limitation of our study is the inability to track changes in patient expectations over time, as our dataset lacks longitudinal data. Future research could address this gap by prospectively following patients to capture the dynamics of expectations throughout the follow-up process. Additionally, we contacted only patients who had attended at least one follow-up appointment. Patients who never attended a follow-up appointment might have different expectations and different needs, which we could not detect with this study. The response rate was also relatively low, this could potentially induce bias. For example, responders might have a higher health literacy and greater awareness of the importance of follow-up care. However, we analyzed the demographic characteristics including the follow-up care appointments and could not detect a statistically significant difference between the two groups.

## Conclusion

The low follow-up rates after bariatric surgery are an issue that needs to be improved. Most expectations were related to the content of follow-up care program. Notably, only a few patients perceived a clear link between attending follow-up appointments and achieving better outcomes. Addressing this gap through effective patient education about the important role of follow-up care could enhance appointment attendance and thus improve patient outcomes. Furthermore, assessing and addressing individual patients’ needs during follow-up could further improve patients’ health and outcomes after bariatric surgery.

## Supplementary Information

Below is the link to the electronic supplementary material.

Supplementary file1 (DOCX 15 KB)

Supplementary file2 (DOCX 14 KB)

## Data Availability

The datasets used and/or analysed during the current study are available from the corresponding author on reasonable request.
