# Supplementary material for: Patients’ Expectations and Perspectives on Follow-up Care after Bariatric Surgery in Germany
Source: Obes Surg. 2025 Apr 30;35(6):2174–84. doi: 10.1007/s11695-025-07890-w (PMC12130090; doi:10.1007/s11695-025-07890-w)
Supplement: Supplementary file 1 — Supplementary file1 (DOCX 15 KB) [file 11695_2025_7890_MOESM1_ESM.docx]

Codebook

# Question 1: What are your expectations regarding follow-up care after bariatric surgery?

## Advice (Psychological, Nutrition, Sports, Plastic Surgery, Medication, General)

Advice involves providing information, guidance, and explanations related to one of the categories.

## Support (Psychological, Nutrition, Sports, Plastic Surgery, Support Group, Medication, General)

Support entails assisting in the achievement or action of goals.

## Structural (temporal, personnel, thematic/content-related, respectful communication)

Establishing framework conditions.

## Outcome (positive influencing factors, negative influencing factors)

Improvement of the result.

"That regular monitoring leads to a better outcome."

## Personal experiences (Dissatisfaction, Satisfaction)

## Individual Cases

Not assignable to another category.

# Question 2: What are positive aspects of the follow-up care program?

## Advice (Nutrition, General)

Advice involves providing information, guidance, and explanations related to one of the categories.

## Support (Psychological, Nutrition, Plastic Surgery, General)

Support entails assisting in the achievement or action of goals.

## Structural (temporal, spatial, personnel, thematic/content-related, respectful communication)

Establishing framework conditions.

## Personal experiences (Dissatisfaction, Satisfaction)

## Individual Cases

Not assignable to another category.

# Question 3: What can be improved in the current follow-up care program?

## Advice (Psychological, Nutrition, Sports, Plastic Surgery, Medication, General)

Advice involves providing information, guidance, and explanations related to one of the categories.

## Support (Psychological, Nutrition, Sports, Plastic Surgery, Support Group, Medication, General)

Support entails assisting in the achievement or action of goals.

## Structural (temporal, spatial, personnel, thematic/content-related, communication)

Establishing framework conditions.

## Personal experiences (Dissatisfaction, Satisfaction)

## Individual Cases

Not assignable to another category.

# Question 4: What is currently missing in the program?

## Advice (Psychological, Nutrition, Sports, Plastic Surgery, General)

Advice involves providing information, guidance, and explanations related to one of the categories.

## Support (Psychological, Nutrition, Sports, Plastic Surgery, Support Group, General)

Support entails assisting in the achievement or action of goals.

## Structural (temporal, spatial, personnel, thematic/content-related, communication)

Establishing framework conditions.

## Personal experiences (Dissatisfaction, Satisfaction)

## Individual Cases

Not assignable to another category.
