# Supplementary material for: Patients’ Expectations and Perspectives on Follow-up Care after Bariatric Surgery in Germany
Source: Obes Surg. 2025 Apr 30;35(6):2174–84. doi: 10.1007/s11695-025-07890-w (PMC12130090; doi:10.1007/s11695-025-07890-w)
Supplement: Supplementary file 2 — Supplementary file2 (DOCX 14 KB) [file 11695_2025_7890_MOESM2_ESM.docx]

Examples of the analysis process:

# Example for Question 1:

## Answers:

“*It is important for me to check my blood values to make sure that there are no deficiency symptoms”*

Coding:

Blood test

Deficiency

“*Regular blood tests are very important to me because general practitioners often refuse to conduct such extensive evaluations, as they are not sufficiently familiar with the topic of bariatric surgery”*

Coding:

Blood test

Regularity

Staff (non-general practitioner)

*“Competent medical check-ups at regular intervals and be prepared to answer specific individual questions”*

Coding:

Check-up

Regularity

Individualized care

## Identification of main categories:

All identified topics can be summarized as topics related to the structure of the program.

## Identification of secondary categories:

Secondary topics to the structure include topics related to the content (blood test, check-up, individualized care), related to the time (regularity) and the personal (staff).

# Example for Question 2:

## Answers:

“*Always blood and urine tests and direct referral to nutritional counseling if needed”*

Coding:

Blood test

Nutrition

“*Questioning whether the diet is working and offering nutritional counseling.”*

Coding:

Nutrition

*“That it is checked whether the values are correct”*

Coding:

Blood test

## Identification of main categories:

The topics can be summarized as topics related to the structure and the advice.

## Identification of secondary categories:

Secondary topics to the structure include topics related to the content (blood test, nutrition).

Secondary topics to advice include a topic related to nutrition.

# Example for Question 3:

## Answers:

“*Nutritional counseling.”*

Coding:

Nutrition

*“Individual advice”*

Coding:

General

Individualized care

## Identification of main categories:

All identified topics can be summarized as topics related to advice.

## Identification of secondary categories:

Secondary topics to advice include topics related to nutrition and general advice (individual).

# Example for Question 4:

## Answers:

*“Psychological support”*

Coding:

Support (psychological)

*“Psychological support, assistance with the care of skin folds/dermatological support, support through plastic surgery.”*

Coding:

Support (psychological)

Support (skin care)

Support (plastic surgery)

## Identification of main categories:

All identified topics can be summarized as topics related to support after bariatric surgery.

## Identification of secondary categories:

Secondary topics to the support include topics related to the psychological support and plastic surgery (skin care, plastic surgery).
